# This is the *Age of Microbial Technology*: Crucial roles of learned societies and academies

**DOI:** 10.1111/1751-7915.14450

**Published:** 2024-04-29

**Authors:** Kenneth Timmis, John E. Hallsworth

**Affiliations:** ^1^ Institute for Microbiology Technical University of Braunschweig Braunschweig Germany; ^2^ Institute for Global Food Security, School of Biological Sciences Queen's University Belfast Belfast UK

## Abstract

Microbial technologies constitute a huge and unique potential for confronting major humanitarian and biosphere challenges, especially in the realms of sustainability and providing basic goods and services where they are needed and particularly in low‐resource settings. These technologies are evolving rapidly. Powerful approaches are being developed to create novel products, processes, and circular economies, including new prophylactics and therapies in healthcare, bioelectric systems, and whole‐cell understanding of metabolism that provides novel insights into mechanisms and how they can be utilised for applications. The modulation of microbiomes promises to create important applications and mitigate problems in a number of spheres. Collectively, microbial technologies save millions of lives each year and have the potential, through increased deployment, to save many more. They help restore environmental health, improve soil fertility, enable regenerative agriculture, reduce biodiversity losses, reduce pollution, and mitigate polluted environments. Many microbial technologies may be considered to be ‘healing’ technologies – healing of humans, of other members of the biosphere, and of the environment. This is the *Age of Microbial Technology*. However, the current exploitation of microbial technologies in the service of humanity and planetary health is woefully inadequate and this failing unnecessarily costs many lives and biosphere deterioration. Microbiologists – the practitioners of these healing technologies – have a special, preordained responsibility to promote and increase their deployment for the good of humanity and the planet. To do this effectively – *to actually make a difference* – microbiologists will need to partner with key enablers and gatekeepers, players such as other scientists with essential complementary skills like bioengineering and bioinformatics, politicians, financiers, and captains of industry, international organisations, and the general public. Orchestration and coordination of the establishment and functioning of effective partnerships will best be accomplished by learned societies, their academies, and the international umbrella organisations of learned societies. Effective dedication of players to the tasks at hand will require unstinting support from employers, particularly the heads of institutes of higher education and of research establishments. Humanity and the biosphere are currently facing challenges to their survival not experienced for millennia. Effectively confronting these challenges is existential, and microbiologists and their learned societies have pivotal roles to play: *they must step up and act now*.

## MICROBES ARE SPECIAL

Microbes are the dominant life form on Earth, collectively constituting a global biochemical reactor carrying out an unimaginable number and diversity of reactions that directly or indirectly sustain and regulate all life in the biosphere, and drive planetary processes and evolution (Falkowski et al., 2008; Goldman & Kaçar, 2023; Sousa et al., 2013). These reactions determine to a significant extent the well‐being of the planet and its other biological inhabitants and are at the root of many processes that currently affect humanity, both positively and negatively. They are also the basis of a multitude of biotechnological applications that provide products and services crucial to human survival, development, well‐being, culture, and other endeavours (https://ami‐journals.onlinelibrary.wiley.com/toc/17517915/2017/10/5; Timmis et al., 2017). This makes the discipline of microbiology rather special.

Of course, chemistry also provides a multitude of products and services but, over the span of a few centuries, became increasingly biased towards petrochemistry, driven by the abundance of cheap hydrocarbon feedstocks. However, microbes have been exploring, mastering, optimising, and practising their own chemistry for over a few billion years, exploiting essentially all the diverse feedstocks that the planet provides. Moreover, the chemical catalysts used by chemists must be sourced and disposed of, and sometimes used under extreme conditions – like high temperatures, high pressures, high electric currents, etc. – all of which may engender considerable energetic, economic, and environmental footprints. Traditionally, chemistry has been a linear process, consuming resources and creating products and side products, some of which may be difficult to recycle, and some of which may be toxic and often recalcitrant, so polluting to the environment and prejudicial to the health of impacted organisms (including humans; Fenton et al., 2021). In contrast, microbes as life forms self‐reproduce; generate their own (biodegradable) *bio*catalysts (enzymes); generally manage with low energy and non‐toxic/low toxicity chemical inputs; and can usually recycle the products they create. Microbes are thus not only exceptional transformers of matter and energy but their reactions are also generally environmentally friendly and sustainable, and many of their processes/activities constitute a circular economy; not only a circular ‘biochemistry‐related’ economy (e.g. global nutrient cycles) but also from an anthropocentric viewpoint, a circular monetary economy.

## THIS IS THE *AGE OF MICROBIAL TECHNOLOGY*

**FIGURE 1:**
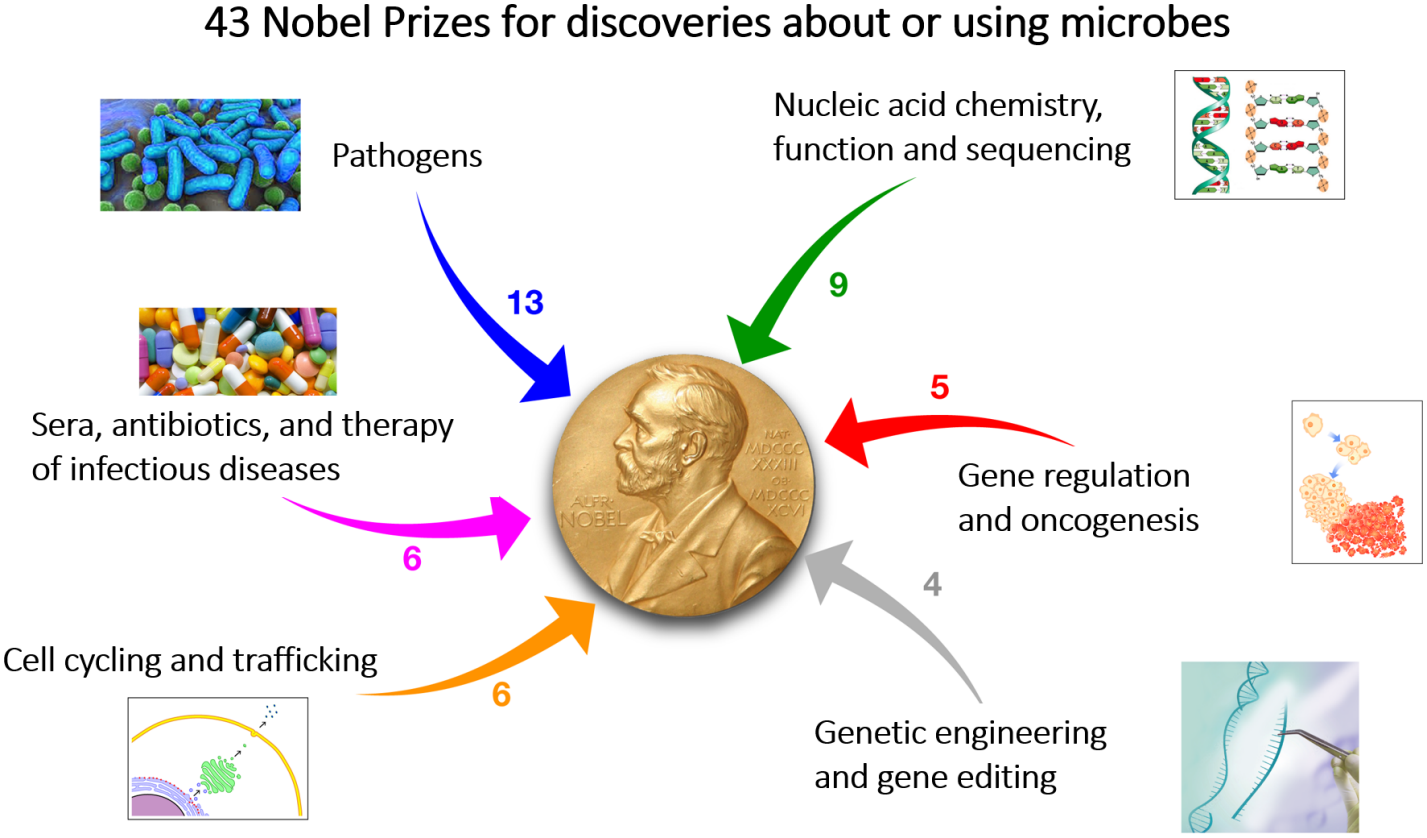
Microbes power scientific discovery: 43 Nobel Prizes. Modified from a figure kindly provided by Rino Rappuoli.

Although the era of Modern Science has been driven by the *Age of Physics* and the *Age of Chemistry*, much of the 20th Century has been the *Age of Microbiology*, characterised by spectacular advances in knowledge obtained by the use of microbes. This includes the elucidation of mechanisms underlying much biochemistry, genetics, the pathogenesis of infectious agents, ecology and food webs, biosphere‐matter transformations and the resulting energy flows, and applications based upon this knowledge, such as the discovery and production of a wealth of pharmaceutical products, including vaccines, that save millions of lives each year, and much more (see Anand et al., 2023). The importance of these discoveries has been recognised by a wealth of Nobel Prizes (Figure 1). Applications resulting from basic research on microbes are not only increasing in number but also in their societal significance (witness the global‐scale deployment of polymerase chain reaction‐based SARS‐CoV‐2 diagnostics, and vaccines, during the recent COVID‐19 pandemic).

As has been discussed, microbial technologies are needed to achieve most, perhaps aspects of all, of the 17 Sustainability Development Goals of the United Nations (see Timmis et al., 2017 and https://ami‐journals.onlinelibrary.wiley.com/toc/17517915/2017/10/5). In the future world (and even other worlds), the ability of humans to survive and flourish will critically depend on microbial technologies, for example in the provision of food, drinking water and healthcare, the recycling of wastes, the recovery and exploitation of natural resources, and so forth (for a more detailed discussion of this, see Anand et al., 2023). Moreover, sustainability on Earth will only be achieved if we learn to live in harmony with, rather than destroying, nature and learn to conserve the biodiversity of the biosphere. For example, the increasing human population, tendency towards smaller households, global warming‐provoked loss of living space, and human displacements and migrations, are driving increased construction of dwellings. The construction industry has a major environmental footprint relating to greenhouse gas production, energy use, a linear (rather than a circular/sustainable) economy, etc., and is an important challenge for sustainability. Facing up to this challenge will involve, inter alia, the exploitation of new microbial technologies to increase built environment sustainability, through the use of the use of bioreceptive materials, microbial fuel cells, and living electronics in building and construction, and reduction in consumption of energy: both ‘embodied’ energy consumed in obtaining, processing and use of construction materials, and ‘operational energy’ needed to provide building services (Armstrong, 2023).

Looking more into the future, there are plans for small‐scale human habitations on Mars in the coming years/decades, and perhaps even cities (Hollander, 2022; McKay et al., 1991). This will only be possible through microbial technologies, not least to create fertile soils, enhance plant health, recycle wastes, and produce sugars via photosynthesis (Koehle et al., 2023; Mapstone et al., 2022). Earth‐based microbiology is also the basis of assessments of habitability (Cockell et al., 2024) that drive life‐detection research and space missions. Microbial activities and biomarkers are therefore central to the search for life on other planets, exploration of other planets for potential habitability, and designing life‐support systems for human space exploration and possible survival on/colonisation of them. This issue is also connected with humanitarian crises on Earth: some envisage relocation of human populations to locations beyond Earth (either Mars or onto spaceships orbiting Earth) to escape from an Earth becoming increasingly hostile to human existence (Musk, 2017; https://www.scientificamerican.com/article/musk‐and‐bezos‐offer‐humanity‐a‐grim‐future‐in‐space‐colonies/). However, the human body – its physiology, immune system, and psychology – has evolved to be suited to Earth conditions (astronaut health deteriorates on space missions), and no other known location has the resources required to sustain a biosphere as rich as we have here on Earth. Therefore, the various applications of microbiology that allow assessment of the habitability of other planets, and that make clear the limitations of space exploration, can serve to increase humanity's urgency to fix the crises currently playing out on Earth. They tell us in no uncertain terms that there is no Planet B, at least among those that are currently known.

These considerations, and others, lead us to view the 21st Century as the beginning of the *Age of Microbial Technology*. Our intention here is not to play off disciplines against one another – quite the contrary – the 21st Century is also characterised by extraordinarily powerful multilateral‐multidisciplinary endeavour and enterprise, so it is also the *Age of Synergistic Multidisciplinarity*. Fundamental disciplines such as physics and chemistry are essential to all of the Sciences (despite the fact that, in many countries, student numbers have declined and many university departments of physics and chemistry have been closed down over recent decades, e.g. https://www.nature.com/articles/nmat1725; https://www.science.org/doi/10.1126/science.314.5804.1363a). Chemists know very well the rules of chemistry/biochemistry which underlie microbial processes; chemical engineers and bioengineers know how to optimise, scale up, and control biotechnological processes, how to feed the microbes, and what to do when the process ends; physicists know about surface and interfacial physics and chemistry, and thus about microbe:surface interactions that influence technical processes; mathematicians and informaticians know how to extract useful information from data gathered, how to model it, and how to use the model to guide the selection of process parameters and to make useful predictions. And the role of the Social Sciences is growing in importance and inter alia brings the voices of key stakeholders into the broader discussions of what society really needs versus what microbiologists think they can and should produce, and integrates biotechnological processes, economics, ethical issues, public perception, and, all‐importantly, policy. Sustainability research is classic interdisciplinary research. But all of this does not dilute the fact that, in terms of what scientists can do to improve the human and planetary condition, microbial applications are currently centre‐stage and we are in the *Age of Microbial Technology*.

## THE EXCEPTIONAL BENEFITS OF MICROBIAL TECHNOLOGIES BRING EXCEPTIONAL RESPONSIBILITIES

Among the sciences, therefore, microbiology is special, which means that microbiologists are special: they collectively have knowledge, expertise, and healing technologies that have the potential to make a wide range of transformational changes benefitting humanity and the biosphere. This is not to imply that microbiologists are inherently gifted people per se: it is simply that their education and training have provided *manna* – knowledge about powerful but (for the most part) invisible agents of change. They see some things (microbial things) that others do not see: microbes that offer solutions/mitigation strategies to problems and crises, and that provide them with the opportunity to orchestrate, or at least propose, actions of immense importance for the well‐being of humanity and the planet. This vision constitutes a gift that brings with it a special duty of care: *the microbiologist's global civic responsibility*.

There are also other sources of responsibility of a more general nature, that include the *Society:Academia Contract* and the *Humanity:Planetary Contract*, which, because of the relevance/importance of microbial technologies, weigh particularly heavily on microbiologists.

## THE SOCIETY:ACADEMIA CONTRACT

Academics are, broadly speaking, fortunate individuals. Despite the daily frustrations of dealing with the diverse bureaucratic and administrative tasks and hurdles of academic life, if we teach well, mentor graduate students and postdocs (who might eventually become professors at prestigious centres of excellence), are successful competing for grant funding for our research projects, and make and publish discoveries that advance human knowledge and understanding of how the world works, and that allow our institutions to make nice press releases (some of which may also pick up traction on social media), we are usually satisfied with the evolution of our professional lives. Microbiologists also travel professionally to beautiful, often exotic places to sample and investigate, and to present results at conferences. We also enjoy *academic freedom* (https://www.unige.ch/international/en/guidelines‐developing‐sustainable‐international‐scientific‐collaborations/academic‐freedom#:~:text=The%20UNESCO%20defines%20academic%20freedom,system%20in%20which%20they%20work%2C). Of course, we may work longer hours than most, especially if we are experimental microbiologists whose experiments dictate our working hours, and, unlike in most other professions, our extra hours usually go unpaid. But, no matter: this does not generally detract from the intellectual rewards we reap.

**FIGURE 2:**
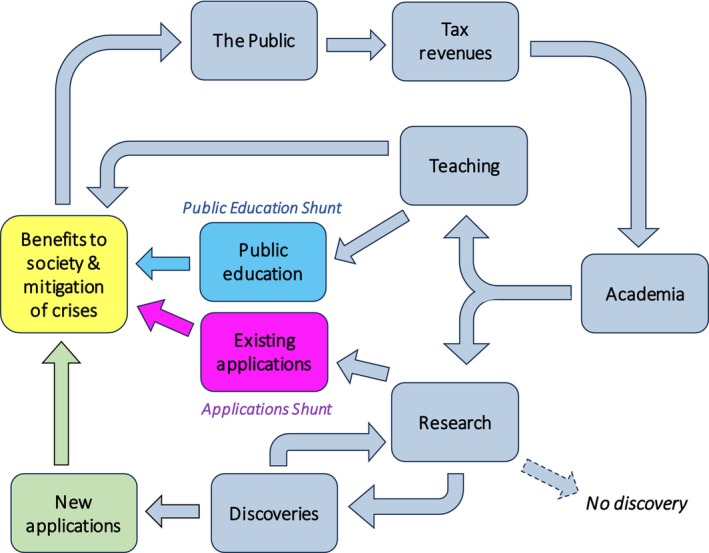
The circular economy of the funding of academia and some of the routes to societal benefits.

Academia is funded to a significant extent by tax revenues. The rationale underlying this allocation of public finances is that it constitutes a vital investment for the future, with an excellent return on investment, represented by research discoveries that lead to applications beneficial to society – including those that can mitigate humanitarian crises – and the education and training of future generations of university graduates and researchers. In effect, this relationship constitutes an unwritten *Society:Academia Contract* and in principle constitutes a circular economy (Figure 2), except for some ‘leakage’/linearity, due to poor science or unfortunate study choices that fail to advance knowledge (dashed arrow in Figure 2). Of course, ‘pure’ (fundamental or basic) science that has no pre‐defined aim for application is both indispensable and transformational, because its discoveries advance the frontiers of knowledge and are therefore the vital enabler and driver of applied developments in science and technology (e.g. (https://www.nigms.nih.gov/education/fact‐sheets/Documents/fact‐sheet‐curiosity‐creates‐cures.pdf); Hallsworth et al., 2023; see also Figure 1). Discoveries immediately leading to beneficial applications are inherently infrequent but ordinarily receive substantial recognition and publicity. The benefits of most discoveries are more usually only realised years later, and their importance is mostly overlooked by the general public. This may lead to an impression that only a small proportion of researchers are contributing to societal benefit. While this is clearly not the case, issues such as questions like ‘and what do you do for a living?’, often trigger a desire to do more that is of obvious direct benefit to society.

There are different mechanisms for academics to increase the immediate and short‐term benefits of their work and two are illustrated in Figure 2. One, the *Applications Shunt* in the research branch, is to explore new, better or additional applications of existing technologies that have a high probability of benefiting society, particularly in areas like sustainability improvement, societal inequality reduction, and climate change mitigation. The other, the *Public Education Shunt* in the teaching branch, is engagement in public education and outreach, which has immediate benefits in improving scientific literacy and understanding of personal and societal issues of importance, and thereby empowering key stakeholders.

## THE HUMANITY:PLANET CONTRACT

The planet and its biosphere provides humanity with most of the goods and services it enjoys. This engenders a fundamental responsibility of humanity to give as well as take, to invest in the environment, to be regenerative, to conserve and not be wasteful, to extract and use the resources sustainably, to live in harmony with nature and not destroy it. This is the *Humanity:Planet Contract*. Microbiologists are again in the front line of responsibility engendered by this contract because microbial technologies are key to many aspects of conservation, recycling, (re)vitalising, healing‐repair, and prophylaxis‐protection (Anand et al., 2023).

## RESPONSIBILITIES OF THE MICROBIOLOGY COMMUNITY

Many of the problems facing communities, nations, humanity in toto, and the planet itself can be either solved or mitigated by microbial processes and interventions (e.g. see Anand et al., 2023). Examples include, but are not limited to, sustainability (https://sdgs.un.org/2030agenda; https://ami‐journals.onlinelibrary.wiley.com/toc/17517915/2017/10/5; Timmis et al., 2017), improving food security at various stages in the supply‐and‐consumption chain (e.g. see Ramos et al., 2023; Timmis & Ramos, 2021; Timmis et al., 2024), and combatting climate change and global warming (Cavicchioli et al., 2019). We know how to do these things, either by modulating microbial activities in nature, or by harnessing specific activities in controlled environments, and many laudable efforts are ongoing worldwide.

But the reality is that humans are not doing enough, and what they are doing is not being done fast enough, so global crises are not being solved. On the contrary, some are worsening (https://news.un.org/en/story/2023/07/1138777; Lenton et al., 2008; Rockström et al., 2009). We urgently need a quantum increase in efforts to harness the power of microbes to tip the balance (Timmis & Hallsworth, 2022). These efforts need to be at all levels: the individual, departments and inter‐departmental groupings, academic disciplines and multi‐ and interdisciplinary networks, the industrial sector, and national and international task forces and organisations. Successful initiatives at all levels usually result from the energy and activity dedicated by talented and motivated individuals and, indeed, there are many examples of ad hoc initiatives involving just a few individuals that are extremely effective in resolving/mitigating important problems (e.g. see https://asm.org/Videos/Microbiology‐Is‐Safe‐Drinking‐Water; http://waterinternational.org; https://earthshotprize.org/winners‐finalists‐listing/).

The triple responsibilities engendered by the *Age of Microbial Technology*, the *Society:Academia Contract* and the *Humanity:Planet Contract* must prompt microbiologists to reflect on the possibility of consecrating greater effort in the two ‘shunts’ of Figure 2. To be clear, we are not arguing for everyone to abandon the work of their lifetime/their passion in favour of humanitarian applications. Moreover, many scientists are overworked, over‐committed, and over‐stressed and thus unable to contribute more. We are simply suggesting that microbiologists individually and as groups take stock and consider what they do and whether they can contribute more to the effort to improve the humanitarian and planetary conditions.

## THE NEED TO PARTNER WITH ENABLERS: ESTABLISHING A COMMON LANGUAGE AND SET OF PRIORITIES

But – while microbial technologies are the *means* of solving or mitigating many problems in many different spheres – *deployment* of the technologies, when and where they are needed, requires political decisions, financing, public acceptance, and, in some cases, public engagement/participation. Additional actors, including scientists beyond the field of microbiology, politicians, economists, lawyers, educators, philosophers, international and non‐governmental organisations, the general public including school children, and more (perhaps, in particular circumstances, even artists, writer‐poets, musicians), can have crucial enabling power. Thus, microbiologists must engage with these key actors, who are not only the enablers of solutions but are also disseminators of relevant microbiology knowledge. Such dissemination is key to the successful implementation of solutions, for increasing appreciation of the potential of microbial technologies, and to identifying neglected (related) problems that also need solving. This engagement requires the use of generally understandable language and terms, as is currently being developed by the International Microbiology Literacy Initiative (IMiLI) for the description of microbial processes and technologies (Timmis et al., 2019, 2024), because fruitful discussions require all players to have a basic level of knowledge and understanding of the issues at stake, and the expertise, mindsets, and language of discussion partners.

While establishing a common language in the short term is both necessary and possible, communication will be much easier and more effective as microbiology outreach, information dissemination via the media and social media, and especially formal education in the general population, increases and the potential of societally‐relevant microbial technologies to solve problems becomes better and more generally appreciated. This requires the creation of microbiology teaching‐outreach resources for the general public. The International Microbiology Literacy Initiative, orchestrated by a huge group of leading international microbiologists, is providing just this and aims to reveal the impact and problem‐solving potential of microbial technologies to societies worldwide (Timmis, 2023; Timmis et al., 2019, 2024).

But a readily‐understood language for, and literacy in, relevant microbiology is not sufficient to ensure the necessary constructive and creative partnering with other enablers. Microbiologists are just people with all the normal range, diversity, and distribution of abilities, skills, and limitations. Thus, although some have the skills and personalities needed to engage with and persuade politicians, economists, the general public, etc., others do not, so the challenge will be to assemble effective teams that represent the necessary spectrum of talents. Many microbiologists, like many other scientists, are rather individualistic, independent people, used to forming alliances with others with whom they share common interests (sometimes doing so through chance encounters). The strategic assembly and deployment of alliances of people with complementary skills and talents to solve global problems is less common. To rectify this deficit, help is needed. This brings us to the importance of microbiological societies, their umbrella organisations, and microbiology academies.

## THE PREORDAINED RESPONSIBILITY OF MICROBIOLOGICAL SOCIETIES, THEIR UMBRELLA ORGANISATIONS, AND MICROBIOLOGY ACADEMIES TO GALVANISE AND MOBILISE DEPLOYMENT OF MICROBIAL TECHNOLOGIES

There are many microbiological societies worldwide; the American Society of Microbiology is one of the largest societies in the life sciences, with 36,000 members (https://asm.org). Most microbiological societies adhere to umbrella organisations such as the International Union of Microbiological Societies (IUMS; https://iums.org), which has 96 national member societies and 26 associate member societies worldwide, and the Federation of European Microbiological Societies (FEMS; https://fems‐microbiology.org), which represents 56 microbiological societies from 40 countries that collectively have 30,000 members. Importantly, the leading microbiologists of a number of some societies are also elected members of academies, such as the European Academy of Microbiology (https://fems‐microbiology.org/european‐academy‐of‐microbiology/) and the American Academy of Microbiology (https://asm.org/academy/academy). The number and range of expertise of the members of these societies and academies are huge, as is their potential. It is vital that this potential be harnessed and focused on solving the problems of human society. In particular, microbiological societies, their umbrella organisations, and academies must progress beyond their normal activities of organising scientific conferences, publishing research journals, and providing grants and awarding prizes, by wielding their organisational potential to engage earnestly and effectively in the orchestration of solving/mitigating humanitarian crises. And microbiology academies – with their collective experience, gravitas, and influence – must lead this endeavour.

The microbiology community and microbiological societies, their umbrella organisations, and academies need to brainstorm and develop concrete plans of action – roadmaps – for which appropriate constellations of talents are assembled to realise the enormous potential of microbiology to solve the major problems faced by humanity. If preventable new catastrophes occur that could have been avoided or mitigated by timely deployment of microbial technologies, the microbiology community will undoubtedly feel culpable, whether or not this is warranted (see also Timmis & Hallsworth, 2022).

## EARTH'S MICROBIOME AS A POLITICAL PARADIGM: THE URGENT NEED FOR MICROBIOLOGY EXPERTS IN POLICY‐DEVELOPMENT UNITS

It may be that most biologists understand that the biosphere, and humanity's future, ultimately depend upon the planet's microbiome. National governments, economists, the industrial sector, and the general public are, however, mostly oblivious to this reality. The microbiomes of humans, other animals, plants, soils, and the wider environment (ecosystems, biomes, landscapes, the biosphere) are not only key to human health but also environmental health including soil fertility and plant productivity, food security, the fate and damage caused by waste and pollutants, mitigating changes in atmosphere composition associated with global warming, producing biofuels and bioplastics to avoid using petroleum, and mitigating other humanitarian crises (e.g. see D'Hondt et al., 2021; Sessitsch et al., 2023; Timmis & Ramos, 2021). Microbiome interventions, and the use of microbes in therapeutic and prophylactic interventions, are the focus of major research efforts (Timmis, Ramos, & Verstraete, 2022; Timmis, Roussilhon, & van de Burgwal, 2022) and show great promise in human healthcare and precision medicine. It is essential, therefore, that national governments and relevant transnational alliances, international organisations – such as the United Nations, the Food and Agriculture Organisation, the United Nations Educational, Scientific and Cultural Organisation, and various aid agencies – have microbiology and microbiome experts embedded as key members of policy development units (see also Salem & Kaltenpoth, 2023).

## CONCLUSION

This is the *Age of Microbiology*: let us as individuals, societies, umbrella organisations, academies, and educational institutions not wait on the sidelines and observe what is happening; let us figure out what needs to happen by whom and get on with the job: carpe diem!

## RECOMMENDATIONS

1. Microbiological societies should identify local‐regional (and international) problems, crises, and opportunities addressable by microbiological technologies of which they collectively have good experience, and set up expert task forces to develop plausible and effective action plans with realistic timetables for achieving the listed actions, i.e. concrete roadmaps. This should involve the identification and engagement of members with connections to key enablers and, in turn, the recruitment of such enablers. This should go beyond discussions; it should also result in commitment and actions. The action plan should include progress monitoring and a retrospective assessment of success measured according to predefined criteria.
2. The same task forces should also consider potential microbial solutions to crises that require further research to become deployable, develop a research plan, and exert influence among research funders to prioritise and fund the recommended research. The need for inter/transdisciplinary approaches should be formulated together with leaders of partner disciplines.
3. Umbrella organisations should similarly address issues of an international and global nature.
4. Academies should take a leading role in spearheading all of these actions, but especially the global ones.
5. Individual microbiologists who have the time and motivation are encouraged to engage in the promotion and deployment of microbial technologies at whatever level they can. They should also consider forming action groups with like‐minded microbiologists and other enablers to get key tasks done. Those in academia should fire up enthusiasm among their students and encourage them to participate in such actions. Individual microbiologists are also the primary players in microbiology outreach (e.g. in schools) and science communication (the general public), so should consider expanding existing/starting new activities (inter alia aided by the availability of teaching resources created by the International Microbiology Literacy Initiative; see Gilmore, 2021).
6. Universities, research organisations and, where appropriate and possible, commercial microbiology operations, should incentivise and actively support their microbiologists to exploit their knowledge and abilities for the good of society, and also encourage/motivate student participation in such activities. Moreover, they should foster the development of civic responsibility in their students by organising courses that reveal societal inequalities and needs, and the potential solutions/mitigating activities.

## FUNDING INFORMATION

No funding information provided.
